# Electrophysiologic testing aids diagnosis and subtyping of myoclonus

**DOI:** 10.1212/WNL.0000000000004996

**Published:** 2018-02-20

**Authors:** Rodi Zutt, Jan W. Elting, Jonathan C. van Zijl, J. Han van der Hoeven, Christiaan M. Roosendaal, Jeannette M. Gelauff, Kathryn J. Peall, Marina A.J. Tijssen

**Affiliations:** From the Department of Neurology (R.Z., J.W.E., J.C.v.Z., J.H.v.d.H., C.M.R., J.M.G., M.A.J.T.), University Medical Center Groningen, University of Groningen, the Netherlands; and Neuroscience and Mental Health Research Institute (K.J.P.), Division of Psychological Medicine and Clinical Neurosciences, School of Medicine, Cardiff University, UK.

## Abstract

**Objective:**

To determine the contribution of electrophysiologic testing in the diagnosis and anatomical classification of myoclonus.

**Methods:**

Participants with a clinical diagnosis of myoclonus were prospectively recruited, each undergoing a videotaped clinical examination and battery of electrophysiologic tests. The diagnosis of myoclonus and its subtype was reviewed after 6 months in the context of the electrophysiologic findings and specialist review of the videotaped clinical examination.

**Results:**

Seventy-two patients with myoclonus were recruited. Initial clinical anatomical classification included 25 patients with cortical myoclonus, 7 with subcortical myoclonus, 2 with spinal myoclonus, and 15 with functional myoclonic jerks. In 23 cases, clinical anatomical classification was not possible because of the complexity of the movement disorder. Electrophysiologic testing was completed in 66, with agreement of myoclonus in 60 (91%) and its subtype in 28 (47%) cases. Subsequent clinical review by a movement disorder specialist agreed with the electrophysiologic findings in 52 of 60; in the remaining 8, electrophysiologic testing was inconclusive.

**Conclusions:**

Electrophysiologic testing is an important additional tool in the diagnosis and anatomical classification of myoclonus, also aiding in decision-making regarding therapeutic management. Further development of testing criteria is necessary to optimize its use in clinical practice.

Myoclonus is a frequently observed hyperkinetic movement disorder, which is often classified according to its anatomical origin: cortical myoclonus (CM), subcortical myoclonus (SCM), spinal myoclonus (SM), peripheral myoclonus (PM), or functional jerks (FJ) in case of a functional movement disorder.

Electrophysiologic testing is frequently useful in distinguishing myoclonus from other hyperkinetic movement disorders, and in identifying its anatomical origin.^1–3^ The tests used in the assessment of myoclonus include polymyography, EEG-EMG back-averaging, coherence analysis, and somatosensory evoked potential (SSEP).^4–8^ Table 1 summarizes the electrophysiologic criteria used in the diagnosis of myoclonus and its subtypes.

**Table 1:**
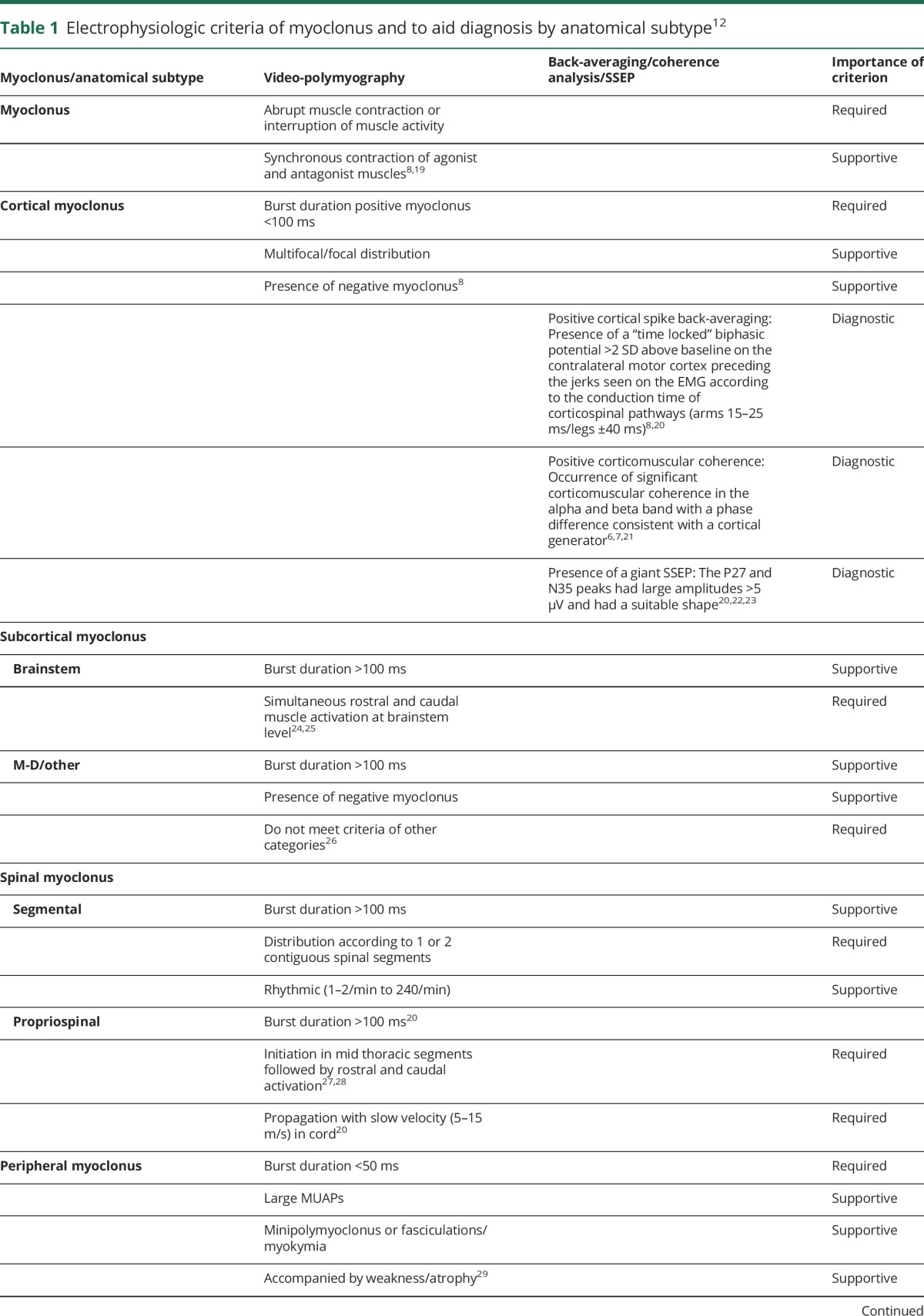

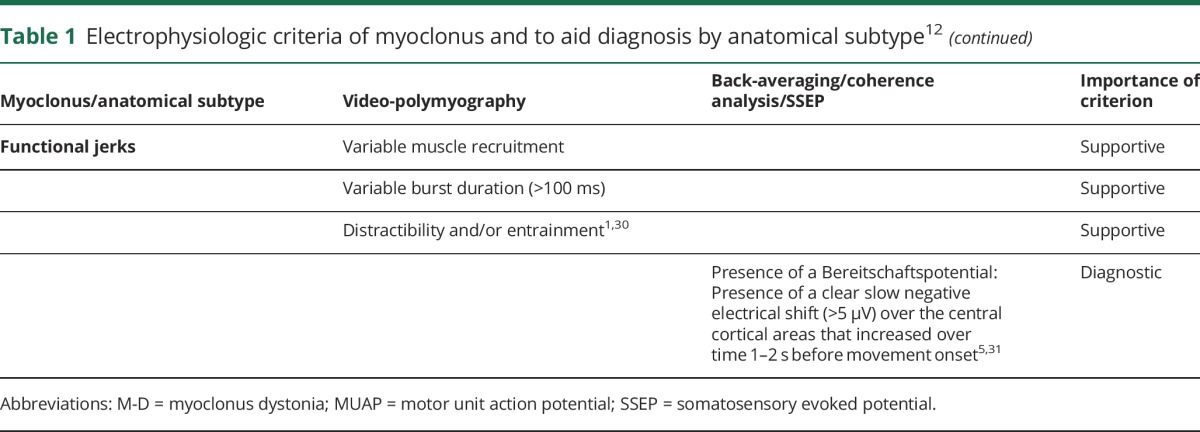
Electrophysiologic criteria of myoclonus and to aid diagnosis by anatomical subtype^12^

The sensitivity and specificity of electrophysiologic testing in patients with myoclonus are largely unknown, with the majority of work to date being limited by small cohorts, highly selected patient populations, or reliance on expert opinion to determine the diagnosis.^9–11^

Our recent retrospective analysis of 85 patients with myoclonus demonstrated the key clinical and electrophysiologic features in distinguishing myoclonus subtypes.^12^ In 74% of cases, the clinical diagnosis of myoclonus was confirmed with electrophysiologic testing, and electrophysiologic assessment of the myoclonus subtype aided diagnosis in 73% of cases. In this study, we sought to apply these principles to a prospectively recruited cohort of patients, evaluating the contribution of electrophysiologic testing in the diagnosis and management of myoclonus.

## Methods

### Participants

Participants with a clinical diagnosis of myoclonus were identified prospectively from inpatient and outpatient settings (July 2014 to June 2016). Exclusion criteria included ongoing inpatient care on the intensive care unit, language and/or literacy barriers, and age 6 years or younger. All participants were followed up for a minimum of 6 months, after which a final diagnosis was made.

### Initial clinical classification

The initial clinical diagnosis of myoclonus and its anatomical subtype was provided by the participants' primary caring neurologist (adult or pediatric), with all participants undergoing a standardized and systematic assessment, including videotaped clinical examination.

**Figure 1:**
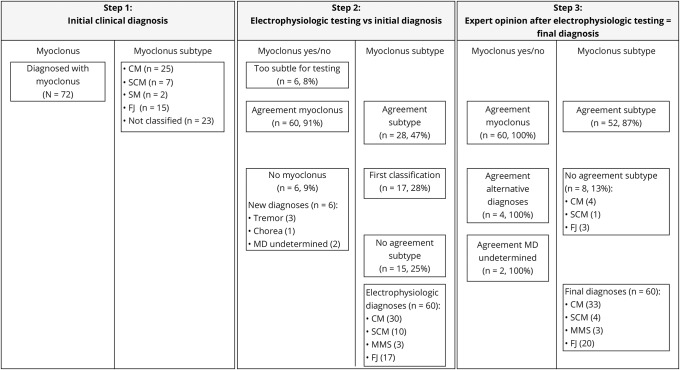
Overview of the stages of clinical assessment and diagnosis undertaken in this study CM = cortical myoclonus; FJ = functional jerks; MD = movement disorder; MMS = multiple myoclonus subtypes; SCM = subcortical myoclonus; SM = spinal myoclonus.

### Electrophysiologic testing

The standardized electrophysiologic protocol included an initial polymyography, with participants excluded at this stage if the myoclonus was too subtle to adequately perform the assessment. For those meeting electrophysiologic criteria for myoclonus, further investigations included EEG-EMG back-averaging (if >25 jerks) or coherence analysis (if jerk frequency was >3 Hz). Where possible, those with CM and SCM underwent testing for SSEPs (figure e-1, http://links.lww.com/WNL/A164).

An experienced neurophysiologist (J.W.E. and J.H.v.d.H.) blinded to the original clinical diagnosis determined whether the findings were consistent with myoclonus, and the likely myoclonus subtype. Table 1 summarizes the electrophysiologic criteria used in determining diagnosis.^12^

### Diagnostic review and 6-month follow-up

A neurologist with expertise in movement disorders (M.A.J.T.), blinded to the initial diagnoses, reviewed the clinical details, videotaped clinical examination, and results of the electrophysiologic testing. Each patient was reviewed again 6 months after their initial assessment to determine any changes to the clinical findings, with the final diagnosis being confirmed by the specialist (figure 1).

### Severity of the myoclonus

The severity of the myoclonus was determined by 2 independent clinicians (R.Z. and J.C.v.Z. or J.M.G.) following review of the videotaped clinical examinations, scoring sections 2 and 4 of the Unified Myoclonus Rating Scale (UMRS), and the 7-point Global Clinical Impression–Severity (GCI-S) scale.

### Power analysis

A power calculation was performed based on our previously reported retrospective analysis.^12^ It was estimated that electrophysiologic testing would support the clinical diagnosis of the myoclonus anatomical subtype in approximately 70%. A change in clinical classification of >20%, due to electrophysiologic testing, was considered clinically relevant. Using the One Proportion Confidence Interval Formula: Exact (Clopper-Pearson), a 95% confidence level, 0.7 (proportion), 0.8 (upper limit), we estimated that a minimum of 56 participants would need to be recruited.

### Statistical analysis

The clinical characteristics were analyzed using Kruskal-Wallis tests for continuous, nonnormally distributed data. Interrater reliability was assessed using the intraclass correlation coefficient (ICC) (2-way mixed, consistency, average measures),^13^ or Cohen κ^14^ where appropriate. A Chi-squared Automatic Interaction Detection (CHAID) (SPSS, IBM, Armonk, NY; parent nodes n < 3, child nodes n > 1) analysis was undertaken to generate a decision tree in order to quantify the importance of the clinical and electrophysiologic criteria in the diagnosis of the myoclonic subtypes.

### Standard protocol approvals, registrations, and patient consents

Full written informed consent was obtained from all participants according to the Declaration of Helsinki. The study protocol was approved by the University Medical Centre Groningen ethics committee (M14.157933, approved July 2, 2014).

## Results

### Overall cohort

A total of 72 patients (32 male; 40 female) were recruited, with a median age of 29 years (range: 7–83 years), 59 from the outpatient setting and 13 from inpatient care.

The demographic details and clinical characteristics of this cohort are summarized in table 2 and table e-1 (http://links.lww.com/WNL/A165), respectively.

**Table 2:**
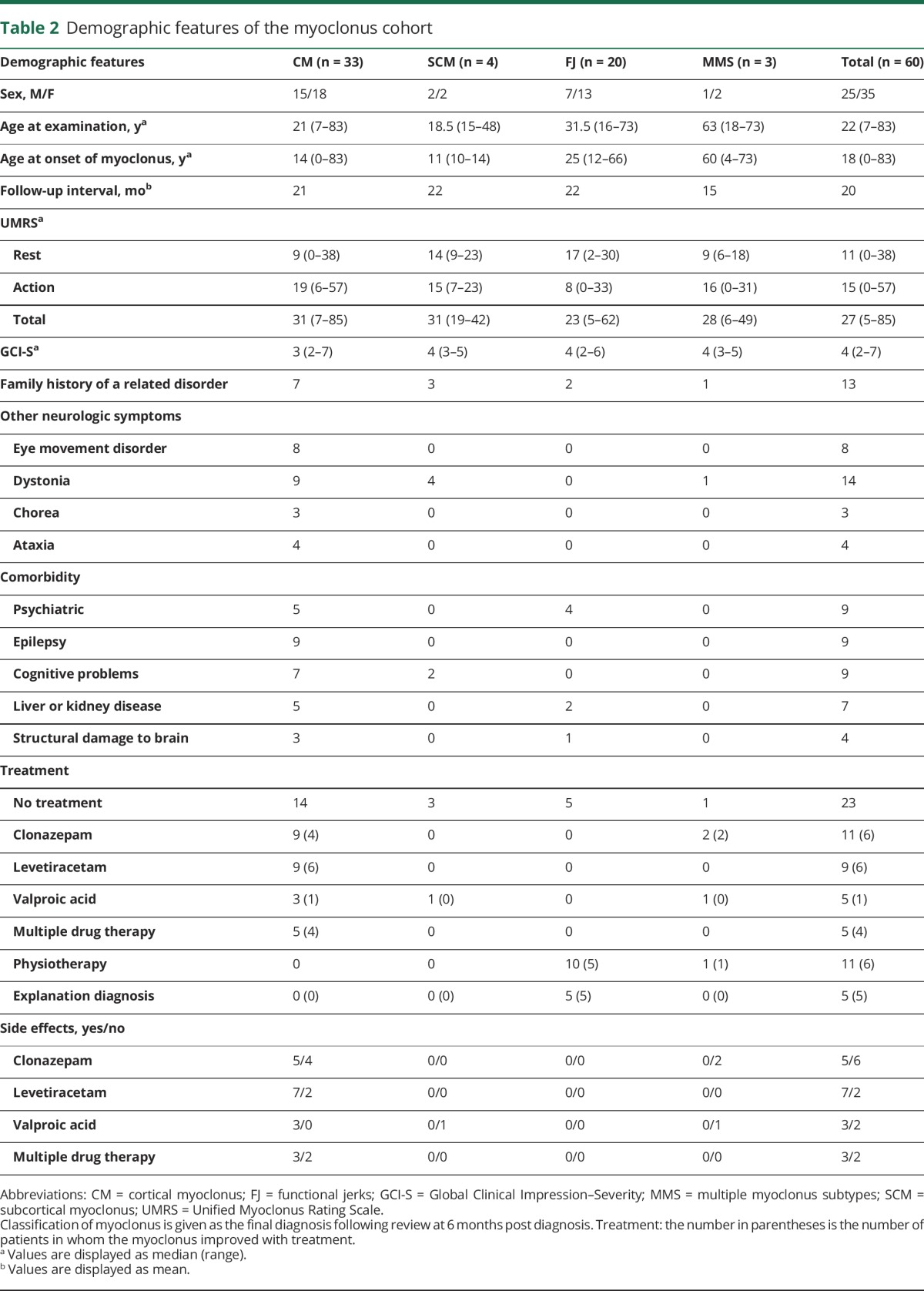
Demographic features of the myoclonus cohort

### Clinical diagnosis of myoclonus pre-electrophysiologic testing

Of the 72 individuals with myoclonus, these were subdivided into CM (n = 25), SCM (n = 7), SM (n = 2), and FJ (n = 15), with subtype diagnoses not possible in 23 patients (32%) because of the complexity of the movement disorder.

### Electrophysiologic diagnoses

In 6 patients (8%), clinically diagnosed with distal multifocal CM, the myoclonic jerks were of such small amplitude that the polymyographic recordings were indeterminate and unable to be interpreted. Of the remaining 66 patients, electrophysiologic testing supported a diagnosis of myoclonus in 60 (91%), with these subdivided into CM (n = 30), SCM (n = 10), multiple myoclonus subtypes (MMS) (n = 3), and FJ (n = 17). A cortical origin was detected in 5 of 9 patients (60%) with CM using back-averaging, and 16 of 20 (80%) using coherence analysis. SSEP analysis demonstrated giant potentials in 3 of 14 patients (21%) with CM, and a Bereitschaftspotential was identified in 5 of 12 patients (42%) with FJ.

A full summary of the electrophysiologic characteristics of this cohort can be seen in table 3.

**Table 3:**
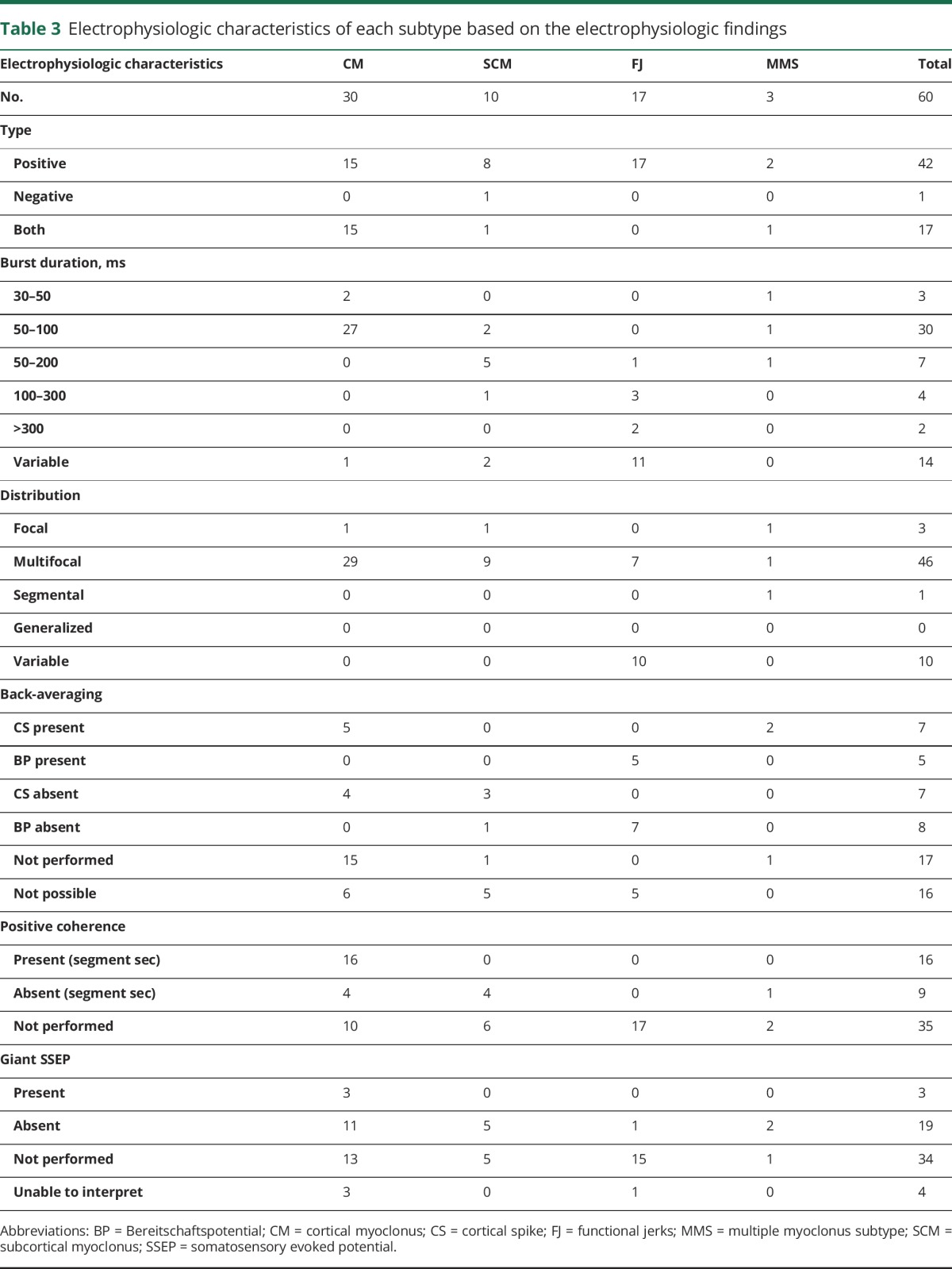
Electrophysiologic characteristics of each subtype based on the electrophysiologic findings

### Comparison of clinical and electrophysiologic diagnoses

There was agreement between the clinical diagnosis and electrophysiologic testing in a diagnosis of myoclonus for 91% (60/66) of the study cohort. Of these 60 cases, there was agreement of its subtype in 28 cases (47%) (14 CM, 2 SCM, and 12 FJ) and disagreement in 15 cases (25%). Of the remaining 17 cases (28%) without a clinical subclassification, electrophysiologic testing proved helpful, subdividing these into 12 CM, 2 SCM, and 3 FJ (table e-2, http://links.lww.com/WNL/A165).

### Clinical opinion of the movement disorder specialist

There was agreement between the electrophysiologic testing and specialist movement disorder opinion in 66 cases, and agreement on its subtype in 52 of 60 cases (87%), considered a “substantial” agreement (κ = 0.78). A summary of the 8 cases in which there was disagreement between expert clinical diagnosis and electrophysiologic testing is provided in table 4; in each, there was a lack of conclusive electrophysiologic findings to facilitate a diagnosis of myoclonus subtype.

**Table 4:**
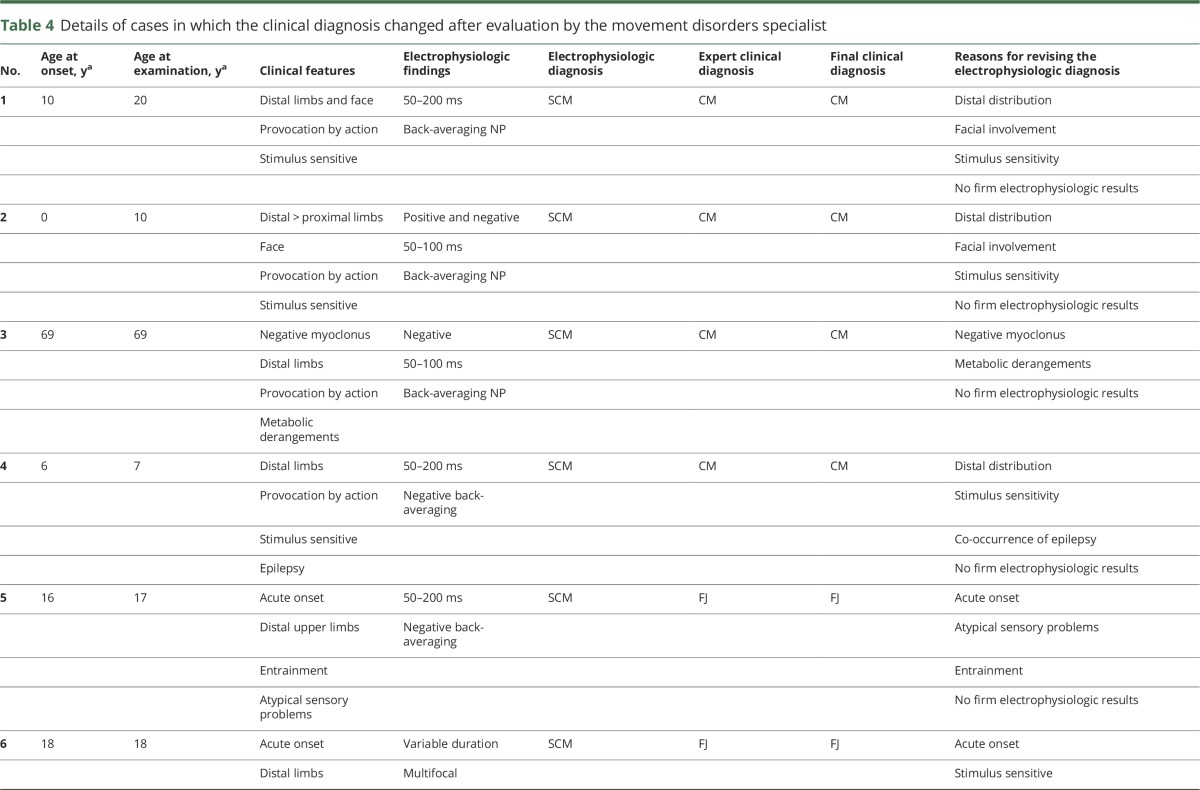

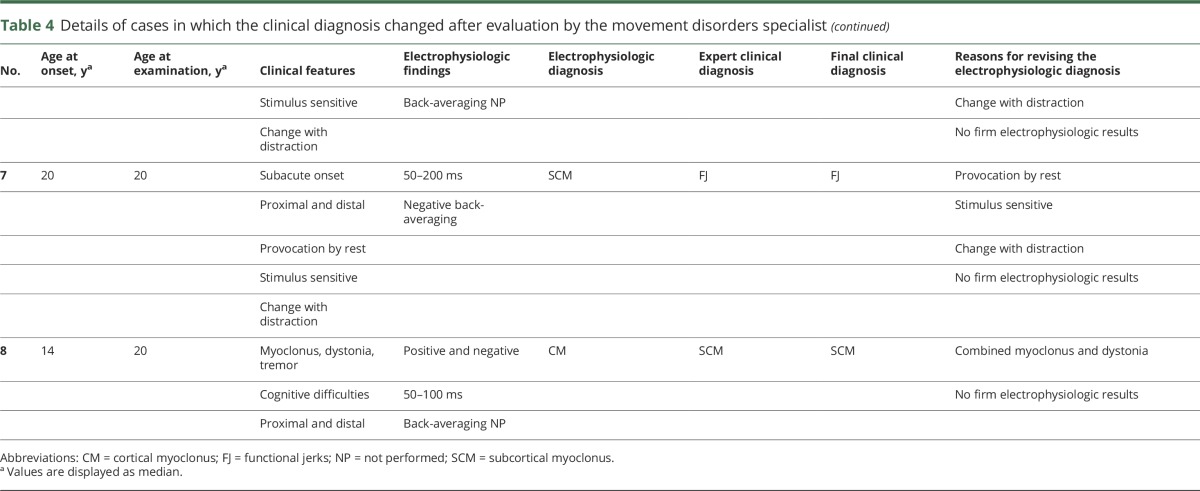
Details of cases in which the clinical diagnosis changed after evaluation by the movement disorders specialist

### Final clinical diagnoses

Follow-up review after 6 months resulted in no changes to clinical diagnosis in all 60 patients, with the final subclassification including 33 CM (55%), 4 SCM (7%), 3 MMS (5%), and 20 FJ (33%). The CHAID analysis demonstrated (1) polymyographic measurement of the myoclonic burst duration, (2) exacerbation of the myoclonus with action, and (3) facial involvement to be the most important criteria in determining myoclonic subtype (figure e-2, http://links.lww.com/WNL/A164).

### Severity of myoclonus

The median UMRS severity score was 27 (Rest 11/128, Action 15/144) and GCI-S score 4/7. No significant statistical difference was observed between the subtypes of myoclonus (*p* = 0.2). The interrater concordance was “excellent” (ICC = 0.94 [95% confidence interval: 0.9–0.96]) and “good” (ICC = 0.72 [95% confidence interval: 0.58–0.82]) for the UMRS and GCI-S, respectively.

### Underlying etiology of the myoclonus

Of the 40 patients diagnosed with an organic movement disorder, an underlying etiology was identified in 21 patients (53%). In 12 patients, a causative genetic mutation was identified, and 9 were found to have an acquired cause including metabolic disturbances (n = 3), drug-induced myoclonus (n = 1), and structural brain lesions (n = 2). Of those with an underlying genetic etiology, the highest rate was among those with CM (n = 10), with mutations in the *NKX2.1* (n = 2) and *NPC1* (n = 2) genes being most common. A single case of a contiguous gene deletion (578 kb, 16p11.2) involving the *PRRT2* gene was identified with an extended phenotype including psychomotor retardation, hemiplegic migraine, epilepsy, myoclonus, and dystonia. All patients with a myoclonic epilepsy syndrome had evidence of epileptiform discharges on EEG, with the CM in those with juvenile myoclonic epilepsy and Lafora disease demonstrating an epileptic origin. All 4 patients with SCM had a clinical diagnosis of myoclonus dystonia, with a *RELN* variant identified in one patient. Table 5 summarizes the etiologic diagnoses and additional clinical characteristics.

**Table 5:**
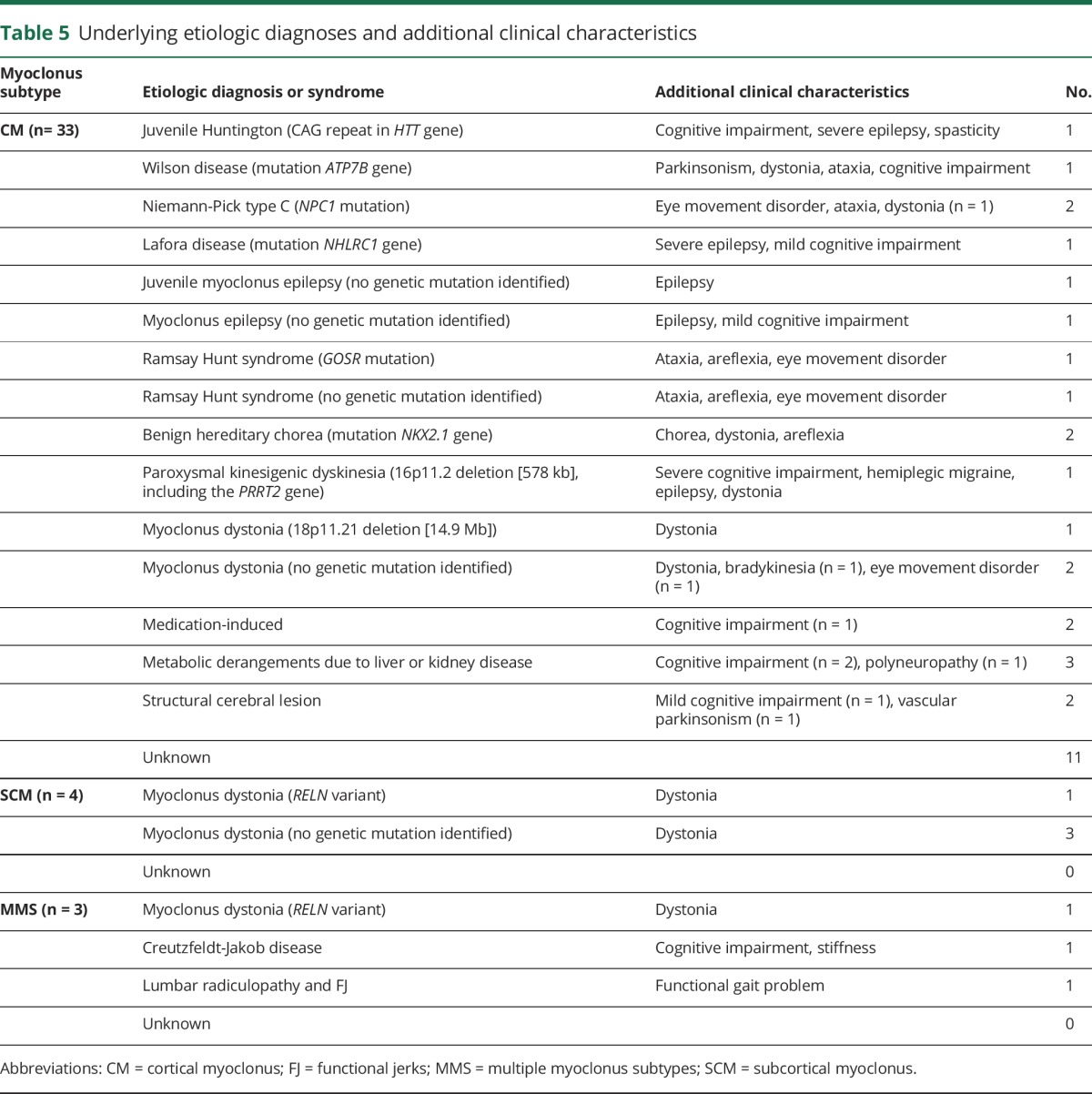
Underlying etiologic diagnoses and additional clinical characteristics

## Discussion

This prospective study has sought to demonstrate the benefit of electrophysiologic testing alongside clinical examination, in determining the diagnosis of myoclonus and its subtype in an unselected cohort. We have shown that this combined approach leads to changes in the initial diagnosis of myoclonus and its subtype in 53% of cases.

Overall, agreement of a diagnosis of myoclonus between the examining clinicians and the electrophysiologic findings was 91% (n = 60), decreasing to 47% (n = 28) with anatomical subtype. These findings contrast with results from similar studies in tremor cohorts (n = 210) where agreement between the 2 assessment forms was 87%, potentially reflecting greater clinical familiarity and larger patient cohorts.^15–17^ We identified several clinical groups in which there was some consistency in the change in diagnosis following electrophysiologic testing. These included those with multifocal myoclonus (principally distinguishing between CM and SCM), combined movement disorders (e.g., myoclonus in the presence of dystonia), and functional jerks. The findings from this study also reflect the difficulty in determining a conclusive clinical diagnosis with myoclonus, and lend weight to the importance of electrophysiologic testing, particularly in nonspecialist centers.

Higher-level electrophysiologic techniques were used to determine whether the myoclonus was of cortical origin or an FJ. The yield of back-averaging and coherence analysis to confirm a cortical origin was 60% and 80%, respectively. The additive value of these techniques was lower than the 100% seen in previous studies, potentially attributable to the heterogeneity of our cohort in contrast to smaller, more selected study groups (n = 20/n = 3).^9,18^ A CHAID analysis demonstrated that a combination of polymyography (burst duration) and clinical phenomenology provided the greatest accuracy (95%) in determining myoclonus subtype.

This study is limited by the lack of a definitive diagnostic test or marker. We have sought to reduce this by ensuring a minimum 6-month follow-up period to allow for any changes in clinical symptomatology. However, this lack of objective testing also serves to reinforce the potential gain of routine electrophysiologic testing to both aid, and provide additional evidence of the diagnosis of myoclonus and its subtype. Our cohort also likely reflects a more complex group of patients than might be expected in routine clinical practice, because of recruitment from a single specialist movement disorder center. We also acknowledge that while the electrophysiologic tests discussed are readily available within our center, such access varies considerably between centers and internationally.

Electrophysiologic testing is an important contributing diagnostic tool for the classification of myoclonus and its subtypes. While this clearly constitutes an important element of clinical work for neurologists with an interest in movement disorders, this algorithm of testing is also likely to be of use for those working in the fields of metabolic disorders, pediatrics, and epilepsy. Further development of the electrophysiologic criteria for myoclonus subtypes, and application of this work to larger, unselected patient cohorts is essential to improve its objectivity and diagnostic value.

## Glossary

CHAID: Chi-squared Automatic Interaction Detection
CM: cortical myoclonus
CS: cortical spike
FJ: functional jerks
GCI-S: Global Clinical Impression–Severity
ICC: intraclass correlation coefficient
MMS: multiple myoclonus subtype
PM: peripheral myoclonus
SCM: subcortical myoclonus
SM: spinal myoclonus
SSEP: somatosensory evoked potential
UMRS: Unified Myoclonus Rating Scale
